# The Impact of Sample Attrition on Longitudinal Learning Diagnosis: A Prolog

**DOI:** 10.3389/fpsyg.2020.01051

**Published:** 2020-06-03

**Authors:** Yanfang Pan, Peida Zhan

**Affiliations:** Department of Psychology, Zhejiang Normal University, Jinhua, China

**Keywords:** cognitive diagnosis, longitudinal learning diagnosis, missing data, sample attrition, Long-DINA model

## Abstract

Missing data are hard to avoid, or even inevitable, in longitudinal learning diagnosis and other longitudinal studies. Sample attrition is one of the most common missing patterns in practice, which refers to students dropping out before the end of the study and not returning. This brief research aims to examine the impact of a common type of sample attrition, namely, individual-level random attrition, on longitudinal learning diagnosis through a simulation study. The results indicate that (1) the recovery of all model parameters decreases with the increase of attrition rate; (2) comparatively speaking, the attrition rate has the greatest influence on diagnostic accuracy, and the least influence on general ability; and (3) a sufficient number of items is one of the necessary conditions to counteract the negative impact of sample attrition.

## Introduction

During the last few decades, to promote student learning, learning diagnosis (Zhan, 2020) or cognitive diagnosis (Leighton and Gierl, 2007) through objectively quantifying the learning status of fine-grained attributes (e.g., knowledge, skills, and cognitive processes) and providing diagnostic feedback has been increasingly valued. Longitudinal learning diagnosis identifies students’ strengths and weaknesses of various attributes throughout a period of time, which also can be seen as an application of learning diagnosis through longitudinal assessments. Longitudinal learning diagnosis not only can be used to diagnose and track students’ growth over time but also can be used to evaluate the effectiveness of diagnostic feedback and corresponding remedial teaching (Tang and Zhan, under review; Wang et al., 2020).

In recent years, to provide theoretical support for longitudinal learning diagnosis, several longitudinal learning diagnosis models (LDMs) have been proposed, which can be divided into two primary categories: the higher-order latent structure-based models (e.g., Huang, 2017; Lee, 2017; Zhan et al., 2019a) and the latent transition analysis-based models (e.g., Li et al., 2016; Kaya and Leite, 2017; Wang et al., 2018; Madison and Bradshaw, 2018). The former estimates the changes in higher-order latent ability over time, and from this, it infers the changes in the lower-order latent attributes. The latter estimates the transition probabilities from one latent class or attribute to another or to the same latent class or attribute. The diagnostic results of these two model types have a high consistency (Lee, 2017). Although the utility of these models has been evaluated by some simulation studies and a few applications, the harm of ubiquitous missing data in longitudinal designs has not yet been considered and studied.

In practice, missing data are hard to avoid, or even inevitable, in longitudinal learning diagnosis and other longitudinal studies. In this current study, we focused on a type of missing data that is common to longitudinal studies, namely, attrition (Little and Rubin, 2020, p. 10). Attrition refers to students dropping out prior to the end of the study and do not return. For instance, in school-level longitudinal learning diagnosis projects, some students may individually drop out before the end of the study because they move to other schools that are inaccessible to the researchers; all students in the class may even drop out altogether because of some unforeseen classroom instructional reasons (see the empirical example in Zhan et al., 2019a).

A higher percentage of attrition at each point in time means the remaining data at subsequent time points provide less diagnostic information, which may also challenge the robustness of measurement models. Some studies have previously employed a complete case analysis that deletes any students who dropped out (e.g., Zhan et al., 2019a). However, this is unfair to those students who were deleted in analysis, because they did not receive any diagnostic feedback. Secondly, it may produce biased results when students with complete data are systematically different from those with missing data. Longitudinal studies are particularly susceptible to such bias, as missing data accumulate over time due to attrition. Therefore, it is necessary to explore the impact of missing data caused by attrition on longitudinal learning diagnosis. This not only helps practitioners better understand the performance of existing longitudinal LDMs in specific test situations with missing data but also provides a reference to psychometricians for future research on the necessity of imputation methods for missing data in longitudinal learning diagnosis. However, as aforementioned, to our knowledge, the harm of ubiquitous missing data in longitudinal designs has not yet been considered and studied in the field of learning diagnosis.

As a prolog, this brief research report aims to explore the impact of various proportions of a common type of attrition (i.e., individual-level random attrition) on longitudinal learning diagnosis through a simulation study. For simplicity and without loss of generality, a simple version of the longitudinal higher-order deterministic-inputs, the noisy “and” gate (sLong-DINA) model (Zhan et al., 2019a) is used in this study. The rest of the paper starts with a brief review of the sLong-DINA model and different types of sample attrition. Subsequently, a simulation study was conducted to mimic the operational scenarios of attrition that may be considered by the sLong-DINA model. Finally, the authors summarize the findings and discuss potential directions for future research.

## Background

### sLong-DINA Model

The sLong-DINA model is one of the representative models of the higher-order latent structural model-based longitudinal LDMs. Compared with the complete version, the special dimensions used to account for local item dependence among anchor items at different time points (see Paek et al., 2014) are ignored in the sLong-DINA model to reduce model complexity and computational burden.

Let *y*_*nit*_ be the response of person *n* (*n* = 1,…, *N*) to item *i* (*i* = 1,…, *I*) at time point *t* (*t* = 1,…, *T*). The sLong-DINA model can be expressed as follows:

First order:

(1) $$\text{logit}\left(P\left(y_{n\,i\,t}=1|\alpha_{n\,t},\gamma_{n\,m},\lambda_{0\,i\,t},\lambda_{1\,i\,t}\right)\right)=\lambda_{0\,i\,t}+\lambda_{1\,i\,t}\prod_{k=1}^{K}\alpha_{n\,k\,t}^{q_{i\,k\,t}}$$

Second order:

(2) $$\text{logit}\left(P\left(\alpha_{n\,k\,t}=1|\theta_{n\,t},{\text{ξ}}_{k},\beta_{k}\right)\right)={\text{ξ}}_{k}\theta_{n\,t}-\beta_{k}$$

Third order:

(3) $$\theta_{n}={\left(\theta_{n\,1},\ldots ,\theta_{n\,T}\right)}^{\prime }∼M\,V\,N_{T}\,\left(\mu ,\Sigma \right)$$

where **α***_*nt*_* = (α*_*n*_*_1_*_*t*_*,…, α*_*nKt*_*)′ denotes person *n*’s attribute profile at time point *t*, α*_*nkt*_*∈{0, 1}, and α*_*n*__*kt*_* = 1 if person *n* masters attribute *k* (*k* = 1,…, *K*) at time point *t* and α*_*n*__*kt*_* = 0 if not; λ_0_*_*it*_* and λ_1_*_*it*_* are the intercept and interaction parameter for item *i* at time point *t*, respectively; *q*_*ikt*_∈{0, 1} is the element in an *I*-by-*K* Q*_*t*_*-matrix at time point *t*, where *q*_*ikt*_ = 1 if item *i* requires attribute *k* at time point *t* and *q*_*ikt*_ = 0 if not; θ*_*nt*_* is person *n*’s general ability at time point *t*; ξ*_*k*_* and β*_*k*_* are the slope and difficulty parameters of attribute *k* at all time points, respectively, because the same latent structure is assumed to be measured at different time points; **μ** = (μ_1_,…, μ*_*T*_*)′ is the mean vector and **Σ** is a variance–covariance matrix:

$$\Sigma =\left[\begin{array}{ccc} \sigma_{1}^{2}\; & & \\ \vdots \; & \ddots \; & \\ \sigma_{1\,T}\; & \cdots \; & \sigma_{T}^{2} \end{array}\right]$$

where σ_1_*_*T*_* is the covariance of the first and *T*th general abilities. As a starting and reference point for subsequent time points, θ*_*n*_*_1_ is constrained to follow a standard normal distribution.

There are two reasons why we did not consider using a general or saturated model (e.g., Huang, 2017; Madison and Bradshaw, 2018). First, general models always need a large sample size to obtain a robust parameter estimate (Jiang and Ma, 2018; Ravand and Robitzsch, 2018). Thus, it is difficult for small-scale educational projects (e.g., school- and classroom-level assessments) to meet this requirement. Second, the parameters in general models are often hard to interpret in practice. Adequate parameter constraints are essential for obtaining interpretable and meaningful insights from the model, which are particularly important in educational and psychological applications to fulfill the need for accountability.

### Sample Attrition

Sample attrition is one of the common sources of missing data in longitudinal studies (Little and Rubin, 2020) and refers to when students drop out prior to the end of the study and do not return. In practice, there are four typical types of sample attrition: individual-level random attrition, class-level random attrition, individual-level nonrandom attrition, and class-level nonrandom attrition. More specifically, (a) the individual-level random attrition reflects the common scenario in which sample size decreased monotonically over time for individual reasons, such as illness, transferring to another school, and reluctance to participate; (b) the class-level random attrition can be seen as an extreme case of individual-level random attrition, where the whole class students drop out for some unpredictable reasons; for example, the testing time may conflict with other course time due to adjusting the curriculum schedule; (c) individual-level nonrandom attrition typically occurs when an individual has achieved a predetermined learning goal, such as mastering the target attributes; thus, some students may feel that there is no need to waste time on follow-up remediation and then quit the follow-up section(s); and (d) class-level nonrandom attrition may occur when the teacher finds that the vast majority of students (e.g., 80%) in the class have mastered the target attributes, then she/he may decide to quit the follow-up section(s) to ensure normal teaching progress. More discussions about sample attrition can be found in Goodman and Blum (1996) and Little and Rubin (2020).

This brief research aims to explore the impact of the individual-level random attrition, which is the simplest type of sample attrition, on longitudinal learning diagnosis. As this is a prolog or preliminary study, we hope that more researchers could continue to study the effects of different types of sample attrition and different types of missing data on longitudinal learning diagnosis (cf., Muthén et al., 2011; Zheng, 2017).

## Simulation Study

### Design and Data Generation

In the simulation study, three factors were manipulated. First, the sample size at the starting time point was varied to be either *N* = 200 or 400 students. According to the national situation in the authors’ country, sample sizes of 200 and 400 translate to approximately 5 and 10 classes with 40 students in each. In real school-level longitudinal learning diagnosis projects, more classes and more students per class are rare. Second, the random attrition rate at each time point (from time point 2) equaled *M*1 = 0% (baseline), 5, 10, 20, 40, and 60% (all the decimal points that might occur in proportional sampling are deleted). The third manipulated variable was test length at each time point at two levels of relatively short (*I*_*t*_ = 15) and relatively long (*I*_*t*_ = 30).

**FIGURE 1:**
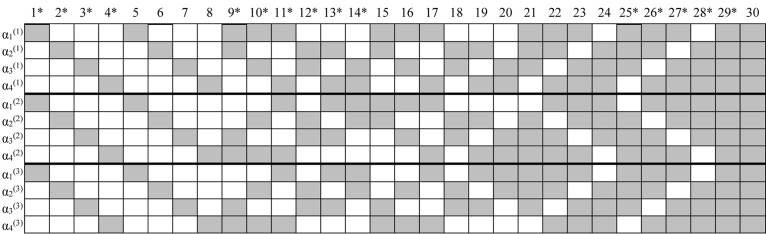
Simulated K-by-I Q′-matrices in simulation study. “*” Denotes items used in the *I* = 15 conditions; gray means “1” and blank means “0”; time point is in parentheses.

According to the authors’ practical experience in longitudinal learning diagnosis (e.g., Tang and Zhan, under review), two or three test times (i.e., one or two sessions of diagnostic feedback and/or remedial teaching) are sufficient for almost all students to master the target fine-grained attributes. Thus, three time points were considered (*T* = 3) in this brief study. In addition, four attributes (*K* = 4) were measured. The first four items for *I*_*t*_ = 15 and the first eight items for *I*_*t*_ = 30, respectively, were used as anchor items. The simulated Q-matrices were presented in Figure 1. In practice, it is common to use high-quality items as anchor items, and thus the anchor item parameters were fixed as λ_0_*_*it*_* = −2.197 and λ_1_*_*it*_* = 4.394. In such a case, the aberrant response (i.e., guessing and slipping) probabilities are approximately equal to 0.1. In addition, the results of Zhan et al. (2019b) indicate that assuming guessing and slipping parameters to follow a negative correlation is more realistic. Thus, non-anchor item parameters were generated from a bivariate normal distribution with a negative correlation coefficient as follows:

$$\left(\begin{array}{c} \lambda_{0\,i\,t}\\ \lambda_{1\,i\,t} \end{array}\right)∼M\,V\,N_{2}\,\left(\left(\begin{array}{c} -2.197\\ 4.394 \end{array}\right),\left(\begin{array}{cc} 1.0\; & -0.6\\ -0.6\; & 1.0 \end{array}\right)\right)$$

This setting leads the guessing and slipping probabilities for all items to follow a positively skewed distribution (mean ≈ 0.1, minimum ≈ 0.01, and maximum ≈ 0.6). Attribute slope parameters were fixed at ξ_*k*_ = 1.5 for all attributes, and attribute difficulty parameters were fixed at β = (−1, −0.5, 0.5, 1). For the general abilities on different time points, the correlations among them were set as 0.9. Between two consecutive time points, the overall mean growths were set at 1, and the overall scale changes were set at $\sqrt{1.25}$.

Furthermore, the response data without attrition (i.e., *M*1 = 0%) were generated from the sLong-DINA model based on the above-generated parameters. For the response data with attrition, a different proportion of students were randomly sampled as attrition from time point 2. Then, these selected students’ responses were modified as missing (i.e., NA), and students who had been drawn out did not appear in the subsequent section(s). In other words, some students were dropped out from time point 2, while some others were dropped out until time point 3. The data were generated by using R software, and the data generation code is available from the authors.

### Analysis

In this brief study, the parameters of the sLong-DINA model are estimated using the Bayesian Markov chain Monte Carlo method via Just Another Gibbs Sampler (JAGS) software. The prior distribution of the model parameters and the corresponding JAGS code are displayed in Supplementary Table S1 in the online supporting materials. More details about how to use the JAGS code for Bayesian CDM estimation can be found in a tutorial by Zhan et al. (2019c).

Thirty replications were implemented in each condition. For each replication, two Markov chains with random starting points were used and 15,000 iterations were run for each chain. The first 10,000 iterations in each chain were discarded as burn-in. Finally, the remaining 10,000 iterations were used for the model parameter inferences. The potential scale reduction factor (PSRF; Brooks and Gelman, 1998) was computed to assess the convergence of each parameter. Values of PSRF less than 1.1 or 1.2 indicate convergence. The results indicated that PSRF was generally less than 1.1, suggesting acceptable convergence for the setting specified.

To evaluate parameter recovery, the bias and the root mean square error (RMSE) were computed as $\text{bias}\,\left(\hat{v}\right)=\sum_{r=1}^{R}\frac{{\hat{v}}_{r}-v}{R}$ and $\text{RMSE}\,\left(\hat{v}\right)=\sqrt{\sum_{r=1}^{R}\frac{{\left({\hat{v}}_{r}-v\right)}^{2}}{R}}$, where $\hat{v}$ and *v* are the estimated and true values of the model parameters, respectively; *R* is the total number of replications. In addition, the correlation between the true values and estimated values (Cor) for some parameters (e.g., general abilities) were computed to evaluate the recovery. For attribute recovery, the attribute and pattern correct classification rate (i.e., ACCR and PCCR) were computed to evaluate the classification accuracy of individual attributes and profiles: $\text{ACCR}=\sum_{r=1}^{R}\sum_{n=1}^{N}\text{I}\left({\hat{\alpha }}_{n\,k\,r}=\alpha_{n\,k\,r}\right)/N\,R$ and $\text{PCCR}=\sum_{r=1}^{R}\sum_{n=1}^{N}\text{I}\left({\hat{\alpha }}_{n\,r}=\alpha_{n\,r}\right)/N\,R$, where I(•) is an indicator function. In reference to Zhan et al. (2019a), two kinds of PCCR were considered in this brief research, namely, the PCCR and the Longitudinal PCCR. The former focuses on whether *K* attributes can be correctly recovered at a given time point, while the latter focuses on whether all *TK* attributes can be correctly recovered (e.g., if *T* = 3, the pattern contains 12 attributes).

## Results

**FIGURE 2:**
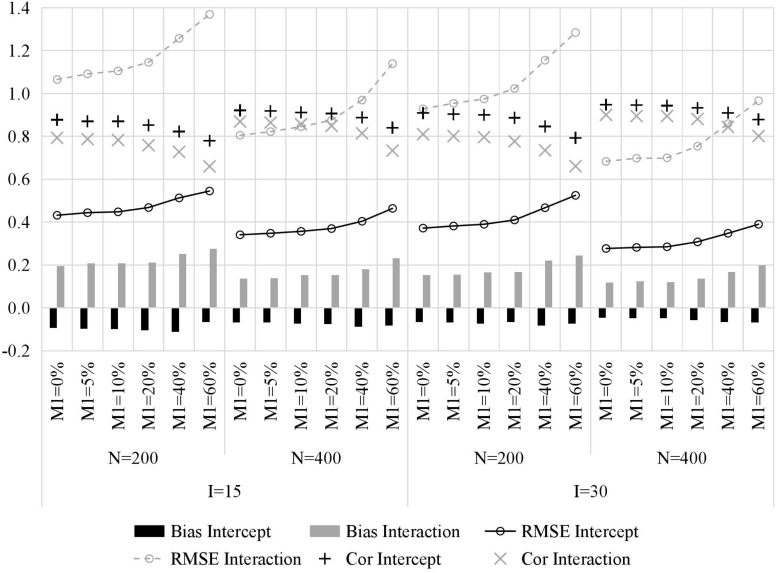
The recovery of item parameters in simulation study. M1, attrition rate; N, sample size; I, test length; Bias, mean bias across all items; RMSE, mean root mean square error across all items; Intercept, item intercept parameter; Interaction, item interaction.

Figure 2 presents the recovery of item parameters. First, one of the most important results is that, with the increase of the attrition rate, the recovery of item parameters decreases, which manifests as larger bias, higher RMSE, and lower Cor. Second, increasing the number of classes (i.e., sample size) and test length yields better recovery of item parameters, and the former is more influential. Third, intercept parameters were generally estimated more accurately than interaction parameters, mainly because the number of individuals who mastered all required attributes is typically less than the number of individuals who do not master all required attributes.

**FIGURE 3:**
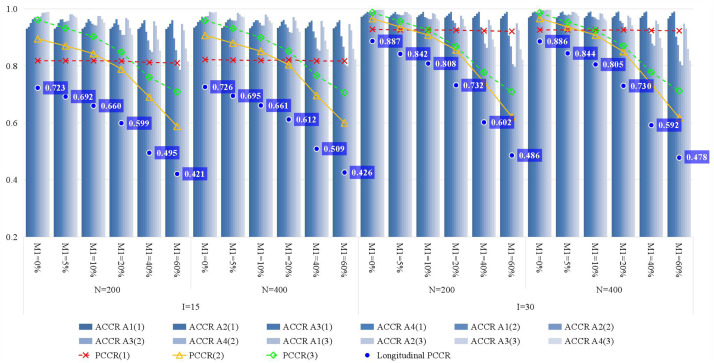
The recovery of attributes in simulation study. M1, attrition rate; N, sample size; I, test length; ACCR, attribute correct classification rate; PCCR, pattern correct classification rate; time point is in parentheses.

Figure 3 presents the recovery of attributes. With the increase of the attrition rate, the classification accuracy quickly decreases, particularly for the Longitudinal PCCR. Since there is no attrition at time point 1, the PCCR of time point 1 is primarily affected by test length. Then, for the PCCR of time points 2 and 3, their downward trend is almost consistent with that of the Longitudinal PCCR. Therefore, if the PCCR is maintained above 80% and the Longitudinal PCCR is maintained above 60%, an attrition rate of less than or equal to around 20% and around 40% is acceptable for short tests and long tests, respectively. In addition, there is a significant result that deserves attention, which is that the classification accuracy of time point 3 is better than that of time point 2; this was also found in the study of Zhan et al. (2019a). Although we currently do not know how to interpret this phenomenon, it is at least not negative for longitudinal learning diagnosis. Furthermore, increasing the number of classes and test length yields higher classification accuracy, but the former has a limited effect.

**FIGURE 4:**
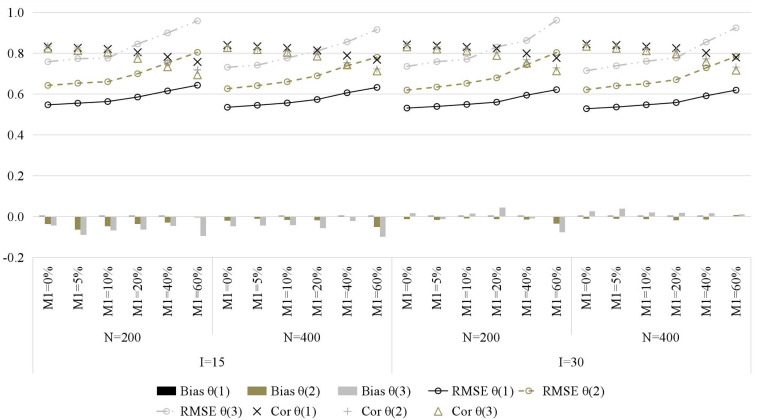
The recovery of general ability parameters in simulation study. M1, attrition rate; N, sample size; I, test length; Bias, mean bias across all persons; RMSE, mean root mean square error across all persons; Cor, correlation between generated and estimated values; time point is in parentheses.

Figure 4 presents the recovery of general ability parameters. Similarly, with the increase of the attrition rate, the recovery of general ability parameters gradually decreases, which is manifested as higher RMSE and lower Cor (bias is less affected). Compared with item parameters and attributes, the attrition rate has less impact on general ability parameters.

## Conclusion and Discussion

This brief research examined the impact of individual-level random attrition on longitudinal learning diagnosis. The results indicate that (1) the recovery of all model parameters decreases with the increase of attrition rate; (2) comparatively speaking, the attrition rate has the greatest influence on the diagnostic accuracy, and the least influence on general ability; and (3) a sufficient number of items is one of the necessary conditions to withstand the negative impact of sample attrition. For relatively short tests (e.g., 15 items), a random attrition rate of 20% or less is necessary to achieve an acceptable longitudinal diagnostic accuracy (i.e., longitudinal PCCR > 0.6); conversely, for relatively long tests (e.g., 30 items), a random attrition rate of 40% or less is necessary.

In summation, the results of this brief study have demonstrated that sample attrition or missing data have a significant impact on diagnostic accuracy of longitudinal learning diagnosis. Therefore, the topics of sample attrition and missing data are worth studying in longitudinal learning diagnosis. As a prolog to future research, the current study only considered some simple cases and left many issues for further discussion. First, this brief research only explores the impact of sample attrition on the sLong-DINA model. Whether the conclusions apply to other longitudinal LDMs is still worth further study in the future. Second, in a different manner from attrition that was focused on this brief research (i.e., monotone missing pattern), a student can be missing at one follow-up time and then measured again at one of the next, resulting in a non-monotone missing pattern. Students’ returning indicates that more information is contained in the data. Thus, it can be inferred that the negative influence of the non-monotone missing pattern on longitudinal learning diagnosis is less than that of attrition. However, the specific degree of its impact remains to be determined. Third, the number of simulation conditions in this brief study is still limited. More independent variables (e.g., the number of attributes and the attribute hierarchies) and more complex test situations (e.g., more time points) can be considered in future studies to provide more reference information for practitioners.

Fourth, in practice, students are nested in classes, and classes are further nested in schools. Such a multilevel data structure is not considered in the current study. By utilizing multilevel LDMs (e.g., Huang, 2017; Wang and Qiu, 2019) in future research, the multilevel data structure can be considered and the impact of class-level attrition can also be studied. Fifth, similar to the Andersen’s longitudinal Rasch model (Andersen, 1985), for general ability, the sLong-DINA model focuses on the estimates at different time points rather than a specific growth trend (i.e., linear or non-linear). If practitioners focus on the latter, the growth curve LDMs (Huang, 2017; Lee, 2017) can be used. Sixth, only the individual-level random attrition was considered in this brief study, while the impact of other three types of attrition (i.e., class-level random attrition, individual non-random attrition, and class-level non-random attrition) on longitudinal learning diagnosis still remains to be further studied.

Seventh, in further studies, it would be much more interesting to explore the impact of different missing mechanisms upon the parameter recovery of longitudinal LDMs, instead of just generating data based on the missing completely at random scenario (i.e., random attrition), such as the missing at random with respect to both observed outcomes and covariates and the missing at random with respect to covariates only (Muthén et al., 2011; Zheng, 2017). Eighth, in longitudinal assessments, for meaningful comparisons, it is necessary to ensure that the same construct is measured across time points. In the presence of item parameter drift, a special case of differential item functioning, the interpretation of scores across time points or change scores would not be valid. Thus, the consequences of ignoring item parameter drift in longitudinal learning diagnosis is worthy of further attention (cf., Meade and Wright, 2012; Lee and Cho, 2017). Ninth, in Bayesian estimation, the prior distribution reflects the beliefs of the data analyst. The posterior distribution of model parameters will be affected by their prior distribution, particularly for a small sample size or a limited number of items. The choice of prior distribution is also worthy of attention (da Silva et al., 2018; Jiang and Carter, 2019). In practice, we recommend that the data analyst selects appropriate prior distributions based on the actual situation rather than copy those given in the Supplementary Table S1.

Last but most important, this brief research is only a superficial study of the missing data in longitudinal learning diagnosis. In the broader field of longitudinal studies, methodologists have been studying missing data for decades and have proposed many methods and techniques to address this issue (see, Daniels and Hogan, 2008; Enders, 2010; Young and Johnson, 2015; Little and Rubin, 2020), such as the traditional imputation methods (e.g., arithmetic mean imputation, regression imputation, and similar response pattern imputation), likelihood-based methods, Bayesian iterative simulation methods, and multiple imputation methods. The performance of these methods in longitudinal learning diagnosis is well worth further study.

## Data Availability Statement

The datasets generated for this study are available on request to the corresponding author.

## Author Contributions

YP contributed to manuscript drafting. PZ contributed to the conception, design, data analysis, and revising the manuscript.

## Conflict of Interest

The authors declare that the research was conducted in the absence of any commercial or financial relationships that could be construed as a potential conflict of interest.
