# Supplementary material for: The Impact of Sample Attrition on Longitudinal Learning Diagnosis: A Prolog
Source: Front Psychol. 2020 Jun 3;11:1051. doi: 10.3389/fpsyg.2020.01051 (PMC7325952; doi:10.3389/fpsyg.2020.01051)
Supplement: Supplementary file 1 [file Presentation_1.PDF]

Supplementary Material

Table S1. Sample JAGS code and prior distributions for the sLong-DINA model in simulation study (I = 15)

```
model{
  for(n in 1:N){
    for(i in 1:SI){
      for(k in 1:K){
        w[n,i,k,1] <- pow(alpha[n, k, 1], Q1[i, k])
        w[n,i,k,2] <- pow(alpha[n, k, 2], Q2[i, k])
        w[n,i,k,3] <- pow(alpha[n, k, 3], Q3[i, k])
      }
      eta[n, i, 1] <- prod(w[n, i, 1:K, 1])
      eta[n, i, 2] <- prod(w[n, i, 1:K, 2])
      eta[n, i, 3] <- prod(w[n, i, 1:K, 3])
      logit(p[n, i, 1]) <- lamda0_1[i]+lamdaK_1[i]*eta[n,i,1]
      logit(p[n, i, 2]) <- lamda0_2[i]+lamdaK_2[i]*eta[n,i,2]
      logit(p[n, i, 3]) <- lamda0_3[i]+lamdaK_3[i]*eta[n,i,3]
      Y1[n, i] ~ dbern(p[n, i, 1])
      Y2[n, i] ~ dbern(p[n, i, 2])
      Y3[n, i] ~ dbern(p[n, i, 3])
    }}
  for(n in 1:N){
    for(k in 1:K){
      logit(prob.a[n, k, 1]) <- xi[k] * theta[n,1] - beta[k]
      logit(prob.a[n, k, 2]) <- xi[k] * theta[n,2] - beta[k]
      logit(prob.a[n, k, 3]) <- xi[k] * theta[n,3] - beta[k]
      alpha[n,k,1]~dbern(prob.a[n,k,1])
      alpha[n,k,2]~dbern(prob.a[n,k,2])
      alpha[n,k,3]~dbern(prob.a[n,k,3])
    }}
  for(n in 1:N){theta[n,1:T] ~ dmnorm(mu_theta[1:T], pr_theta[1:T, 1:T])}    #prior of multiple general abilities
  for(k in 1:K){
    beta[k] ~ dnorm(0, 0.25)    #prior of attribute difficulty
    xi[k] ~ dnorm(0, 0.25) T(0, )    #prior of attribute slope
    }
  for(i in 1:SI){
    lamda0_1[i]~dnorm(-2.197, 0.25)    #prior of item intercept
    lamdaK_1[i]~dnorm(4.394, 0.25) T(0, ) #prior of item interaction
  }
  for(i in 1:4){
    lamda0_2[i]<-lamda0_1[i]
    lamdaK_2[i]<-lamdaK_1[i]
  }
  for(i in 5:SI){
    lamda0_2[i]~dnorm(-2.197, 0.25)
    lamdaK_2[i]~dnorm(4.394, 0.25) T(0, )
  }
  for(i in 1:4){
    lamda0_3[i]<-lamda0_1[i]
    lamdaK_3[i]<-lamdaK_1[i]
  }
  for(i in 5:SI){
    lamda0_3[i]~dnorm(-2.197, 0.25)
    lamdaK_3[i]~dnorm(4.394, 0.25) T(0, )
  }
  mu_theta[1] <- 0
  for (t in 2:T){mu_theta[t] ~ dnorm(0, 1)} # hyper prior of the mean of general ability
  L_theta[1, 1] <- 1
  for(tt in 2:T){
    L_theta[tt, tt] ~ dgamma(1, 1)
    for(ttt in 1:(tt-1)){
      L_theta[tt, ttt] ~ dnorm(0, 1)
      L_theta[ttt, tt] <- 0}}
  Sigma_theta <- L_theta %*% t(L_theta)    #hyper prior of the variance-covariance matrix of general ability
  pr_theta[1:T, 1:T] <- inverse(Sigma_theta[1:T, 1:T])}
```
